# Comparative Analysis of Molecular and Serologic Testing for Primary Syphilis: A Population-Based Cohort Study

**DOI:** 10.3389/fcimb.2021.579660

**Published:** 2021-04-23

**Authors:** Caley Bryce Shukalek, Bonita Lee, Sumana Fathima, Angel Chu, Kevin Fonseca, Ranjani Somayaji

**Affiliations:** ^1^ Department of Medicine, University of Calgary, Calgary, AB, Canada; ^2^ O’Brien Institute of Public Health, University of Calgary, Calgary, AB, Canada; ^3^ Alberta Health Services, Edmonton, AB, Canada; ^4^ Department of Community Health Sciences, University of Calgary, Calgary, AB, Canada; ^5^ Department of Pediatrics, University of Alberta, Edmonton, AB, Canada; ^6^ Provincial Laboratory for Public Health, Calgary, AB, Canada; ^7^ Department of Microbiology, Immunology and Infectious Diseases, University of Calgary, Calgary, AB, Canada; ^8^ Snyder Institute for Chronic Disease, University of Calgary, Calgary, AB, Canada

**Keywords:** syphilis, sexually transmitted infections, sexually transmitted diseases (STDs), *treponema pallidum*, molecular diagnostic, anogenital lesions, serology diagnostic

## Abstract

Rising rates of syphilis (*T. pallidum; Tp*) requires rapid diagnosis and treatment to manage the growing epidemic. Syphilis serology is imperfect and requires interpretation of multiple tests while molecular diagnostics allows for potential yes-no identification of highly infective, primary anogenital lesions. Accuracy of this testing modality has thus far been limited to small, highly selective studies. Therefore, we retrospectively assessed a large, adult population of patients with anogenital lesions seen at Sexually Transmitted Infection (STI) clinics in Alberta, Canada who were screened for syphilis and herpes simplex (HSV) 1/2 using PCR to evaluate *Tp-*PCR versus serology to diagnose primary syphilis. 114 (3.1%) of the 3,600 adult patients had at least one *Tp*-PCR+ anogenital lesion with 99 (2.8%) patients having newly positive syphilis serology (new INNO-LIA positive or 4-fold RPR increase). *Tp-*PCR had a sensitivity of 49.3% (95% CI 42.6-56.1) and specificity of 99.9% (99.7-100.0). Positive predictive values and negative predictive values in the study population or when corrected for provincial prevalence were 97.4% (92.5-99.5) or 0.4% (0.4-1.2) and 96.7% (96.1-97.3) or 100.0% (100.0-100.0), respectively. Positive and negative likelihood ratios were estimated at 555 (178-1733) and 0.5 (0.4-0.6), respectively. Review of all *Tp*-PCR performed with or without exclusion of HSV-positive lesions resulted in no significant change in *Tp-*PCR characteristics. Interestingly, 12 of the *Tp-*PCR+ samples had negative serology at time of lesion sampling but became positive within our 28-day testing window. Overall, this study further supports the use of *Tp-*PCR as an accurate assay to rapidly identify, treat, and prevent the spread of primary syphilis.

## Introduction

Syphilis is a bacterial sexually transmitted infection (STI) caused by *Treponema pallidum*. Syphilis can manifest at various stages of infection, and is known as the “great imitator” given its countless clinical presentations (Belda et al., 2009). Its primary presentation is classically as a painless anogenital ulcer. For many years syphilis rates through the general population were very low, measuring less than 1 per 100,000 across Canada (Notifiable Disease Charts, 2020). Starting in 2001, rates have risen sharply to 17.0 per 100,000 persons in 2018 (Interactive Health Data Application (2019); Notifiable Disease Charts, 2020). Alberta’s rates remain well above the national average in recent years, measuring 35.7 per 100,000 persons in 2018 (Canada PHAo, 2019;  Interactive Health Data Application, 2019).

Primary presentations of syphilis can be difficult to diagnose as the painless anogenital ulcer can be confused with both herpes and chancroid (Behets et al., 1999). Notably, the etiologic agent of chancroid, *Haemophilus ducreyi*, is no longer screened for in anogenital ulcer cases due to an absence of cases in Alberta in many years (Wellness AH, 2012). However, rates of herpes simplex virus 1 and 2 (HSV1 and HSV2, respectively) are significantly higher than that of syphilis and the required treatment differs greatly. It is estimated that HSV1 and HSV2 sero-prevalence in Alberta is greater than 50% and ~19%, respectively, contingent on population characteristics (Tilley ASaP, 2008).

Primary syphilitic chancres are highly infectious through skin-to-skin (mucous membrane) contact, making rapid diagnosis and treatment paramount. Patients presenting with an anogenital ulcer are diagnosed primarily on clinical grounds and managed accordingly (Keck, 2005). If clinicians are specially trained and have access to and expertise with a darkfield microscope (DFM), it may be utilized to make near-patient diagnosis of syphilis for specific primary cases (Keck, 2005). However, this technical skill and the required equipment are becoming scarce. Serology testing for non-*Treponema* and *Treponema* antibodies is the primary diagnostic test but can be negative in early primary syphilis, especially if rapid plasma reagin (RPR) is used as a non-Treponema screening test (Binnicker, 2012). For patients with history of syphilis infection, interpretation of results is more complex as *Treponema* antibodies persist after infection, and staging of syphilis infection relies on the results of non-*Treponema* antibodies such as RPR and clinical presentation and findings. Depending on the testing algorithm, syphilis serology can consist of three separate tests (starting with screening test using either *Treponema* antibodies on, e.g., a chemiluminescent enzyme immunoassay or RPR that needs to be quantified in terms of titers if positive, and confirmatory assays). There can be multiple permutations with syphilis serology and must be appropriately interpreted in the clinical context (Laboratories AP, 2016). Anogenital ulcers can thus be diagnostically challenging and use of clinical findings and laboratory testing in combination is critical.

More recently, testing for *T. pallidum* by polymerase chain reaction (*Tp-*PCR) on suspected primary lesions has been approved as one of the US Centers for Disease Control (CDC) recommended tests (CDC AoPHL, 2009). Syphilis PCR is advantageous as it allows for molecular testing of an ulcer to confirm the presence of the spirochete bacteria not discernible by clinical evaluation alone, and does not require the presence of antibodies, or large numbers of bacteria as for microscopy (Heymans et al., 2010; Whitfield et al., 2010). A number of small trials (N=30, *n*=1516) have demonstrated pooled *Tp*-PCR testing to have relatively high sensitivity (78.4, 95% CI 68.2-86.0) and specificity (96.6, 95% CI 95.5-97.5) in relation to clinical diagnosis of primary syphilis, and be superior to serology given the presence of a window period for antibodies development especially in high risk populations (Palmer et al., 2003; Leslie et al., 2007; Gayet-Ageron et al., 2009; Heymans et al., 2010; Whitfield et al., 2010; Shields et al., 2012). A meta-analysis of PCR testing characteristics has suggested the likelihood ratios of a positive (21.0, 95% CI 15.5-28.4) or negative (0.27, 95% CI.018-0.40) result are diagnostically useful (Gayet-Ageron et al., 2013). However, no large-scale population-based studies of PCR utility have been performed. Thus, using laboratory data from clinically suspicious cases we aimed to independently evaluate the comparative ability of PCR testing to serology for the diagnosis of primary syphilis using a population-based approach.

## Methods

### Sample Selection

A retrospective population-based study of two large STI expert centers was conducted in the western Canadian province of Alberta. Syphilis PCR and serology test results obtained from adult patients (≥18 years) who had at least one specimen submitted for syphilis PCR presumably due to the presence of anogenital ulcer at the STI clinics in the two largest cities, Edmonton and Calgary, Alberta, from Jan 1, 2007 until Dec 31, 2014 were included in our cohort analysis. Suspected primary syphilis ulcers were identified by experienced STI clinic nurses and swabbed for testing that includes syphilis and HSV 1 and 2. The results of *Tp*-PCR testing for primary syphilis compared to syphilis serology were analyzed to determine the testing characteristics and validity of *Tp*-PCR testing. Results of HSV 1 and 2 PCR testing typically performed simultaneously were included allowing for differentiation of lesions or co-infection.

### Syphilis Diagnostic Test Methods

All *Tp*-PCR tests in Alberta were performed at the Provincial Laboratory for Public Health (ProvLab) sites in Alberta, Canada and based on pre-existing protocols (Koek et al., 2006; Chen et al., 2006). *Tp*-PCR testing involves amplification of DNA sequences from two different target genes, polA and tpp47; both of these being highly conserved in the *Treponema* genus (Burstain et al., 1991; Liu et al., 2001). Detection of both of these target genes through amplification denoting a positive result after specimen collection is performed by trained nurses at either STI clinic according to procedures outlined (Services AH, 2019). Serologic testing, also performed at ProvLab according to internal protocols, was performed using syphilis enzyme immunoassay (EIA) looking for IgM and IgG antibodies specific for *T. pallidum* (Architect Syphilis TP Chemiluminescent Microparticle Immunoassay, Abbott Laboratories, Abbott Park, IL) as the screening test, followed by RPR (Macro-Vue RPR Card Tests, Brewer Diagnostic Kits, Becton Dickinson Microbiology Systems, Franklin Lakes NJ) and confirmatory treponemal test, a line immunoassay (INNO-LIA Syphilis, Innogenetics NV, Ghent, Belgium), if EIA was indeterminate or positive. Newly active infection by serology was counted by either a new positive by INNO-LIA testing or with a 4-fold increase in their RPR titer from the most recent result (Ratnam, 2005). Syphilis serology is considered negative when the EIA is negative (no further test); when the EIA is indeterminate or positive but the INNO-LIA is negative and there is no detection or a less than 4-fold increase in the RPR; or the EIA and INNO-LIA are persistently positive from previous measures and there is no detection or a less than 4-fold increase in the RPR. HSV 1 and 2 PCR testing at the ProvLab is also based on pre-existing protocols (Wong et al., 2016).

### Ethics

This study was approved by the Conjoint Health Research Ethics Board (CHREB), Alberta Health Services and University of Calgary (REB14-2126).

### Statistical Analysis

Data was arranged into cases based around *Tp*-PCR, any syphilis serology tests performed within a window period (-7 to +28 days), and HSV PCR, if available, then anonymized and analyzed in aggregate form. Data analysis included determination of test characteristics (i.e. sensitivity, specificity, positive predictive value [PPV], and negative predictive value [NPV]) of *Tp*-PCR testing in comparison with syphilis serology test results in the diagnosis of primary syphilis. Serology provided true positive and negative results as the relative, gold standard. Sensitivity was estimated as the percent of true positive (serology) divided by the sum of true positive and false negative. Specificity was estimated as the percent of true negative (serology) divided by the sum of true negative and false positive (Tp-PCR). Positive and Negative likelihood ratios (LRs) were estimated from the calculated sensitivity and specificity, as previously described (McGee, 2002). Local epidemiological data was used to determine both PPV and NPV by Bayes Theorem (Wellness AH, 2019). Statistical analysis was performed using Stata v16.1 (College Station, Texas).

## Results

### Syphilis PCR (*Tp*-PCR) Testing Demographics and Positivity

A total of 3,871 unique adult patients had at least one *Tp*-PCR test sampled, of which 254 (6.6%) patients had no syphilis serology within the allotted window period and 17 (0.4%) patients had *Tp*-PCR testing cancelled. Of the remaining 3,600 patients, there were 1,865 males (51.6%) with an average age of 30.6 ± 10.6 years (**Table 1**). The average age of those with any a *Tp*-PCR result was significantly higher at 35.0 ± 12.8 years (p-value <0.01). There were 1.2 ± 0.4 cases with *Tp*-PCR testing completed among all patients with significantly lower (1.1 ± 0.4, p-value <0.01) and higher (1.5 ± 0.8, p-value <0.01) average of cases with testing in persons with all negative or any positive *Tp*-PCR result, respectively. Excluding patients who were positive for HSV1 or HSV2, there were 1,119 (54.1%) males with average age of 30.8 ± 11.2 years, which was significantly different than all *Tp*-PCR results in all patients (p-value <0.01). Similarly, patients among this HSV1 & HSV2 negative population with any positive *Tp*-PCR were older and had more cases with *Tp*-PCR testing (**Table 1**).

**Table 1:** Patient characteristics all and HSV1/2 negative persons screened with *Tp*-PCR for anogenital lesions.

ALL PATIENTS (1 sample/patient)	All *Tp*-PCR (%)	Negative *Tp*-PCR (%)	Positive *Tp*-PCR (%)
	*n=3600*	*n=3486*	*n=114*
Male	1851 (51.4)	1762 (50.5)	89 (78.1)
Age	30.6 ± 10.6	29.6 ± 10.5	35.0 ± 12.8*
Number of independent PCR tests	1.2 ± 0.4	1.1 ± 0.4*	1.5 ± 0.8*
** HSV1 & HSV2 NEGATIVE PATIENTS** ** (1 sample/patient)**	**All *Tp*-PCR (%)**	**Negative *Tp*-PCR (%)**	**Positive *Tp*-PCR (%)**
	*n=2068*	*n=1963*	*n=105*
Male	1119 (54.1)	1039 (52.9)	80 (76.2)
Age	30.8 ± 11.2*	30.5 ± 11.0	35.2 ± 13.0*^$^
Number of independent PCR tests	1.1 ± 0.4*	1.1 ± 0.4*	1.4 ± 0.8*^$^

*significant difference from all Tp-PCR results in all patients, denoted by p-value < 0.05.

^$^significant difference from all Tp-PCR results in HSV1 & HSV2 negative patients, denoted by p-value < 0.05.

### Syphilis PCR (*Tp*-PCR) Testing Results

Using all cases for the 3,600 patients included, there were 4,157 lesions sampled, at discrete timepoints, in which *Tp*-PCR was collected with serology in the window period. Of the 4,157 *Tp*-PCR assays completed, 168 were positive (**Supplementary Table 1A**). Of these, 114 were positive on first anogenital lesions assessment with an additional 54 cases positive on a remote, secondary presentation. Of these, 101 were newly positive EIA and INNO-LIA, while 64 of these cases had previously positive EIA and INNO-LIA but were note to have a 4-fold or greater increase in RPR. Three of these *Tp*-PCR positive cases were serology negative. 136 *Tp*-PCR negative lesions had positive serology by INNO-LIA or increased RPR titer within the window period. 3,853 patient cases had both negative *Tp*-PCR and serology. There were 1,787 sampled lesions that were positive for HSV1 or HSV2. Excluding these samples, 149 were *Tp*-PCR and serology positive, 3 were *Tp*-PCR positive and serology negative, 126 met criteria for serology consistent with reinfection and had negative *Tp*-PCR, and 2,095 were negative for both (**Supplementary Table 1B**).

Assessing only the first case of all 3,600 patients, there were 114 (3.1%) positive samples for *Tp*-PCR of which 111 had positive serology reflecting a first time active or syphilis reinfection and three had negative serology (**Supplementary Table 1C**). Of these, 91 had the first recorded positive EIA, INNO-LIA, and RPR, 10 had prior negative serology that changed to positive EIA and INNO-LIA without reactive RPR, and 10 had previously recorded positive EIA and INNO-LIA results but a 4-fold or greater increase in RPR, from previous stable or undetectable RPR, denoting reinfection. Twelve samples that were initially negative by serology – collected on the same day as the *Tp*-PCR specimen – and later became positive by serology as denoted by newly positive INNO-LIA, or, if previously positive, a 4-fold increase in RPR. Positive syphilis serology, as outlined, but negative by *Tp*-PCR was seen in 114 patients. Of these, 41 had the first recorded positive EIA, INNO-LIA, and RPR, one had prior negative serology that changed to positive EIA and INNO-LIA without reactive RPR, and 72 had previously recorded positive EIA and INNO-LIA results but a 4-fold or greater increase in RPR, from previous stable or undetectable RPR, denoting reinfection by serology. The remaining 3,372 cases had both negative *Tp*-PCR and serology. Excluding the 1,532 samples that were positive for HSV1 or HSV2, there were 102 *Tp*-PCR and serology positive, three *Tp*-PCR positive and serology negative, 105 *Tp*-PCR negative and serology positive, and 1,858 *Tp*-PCR and serology negative assessments (**Supplementary Table 1D**).

Of the 254 patients without syphilis serology within the window period, 1 *Tp*-PCR result was positive. Of the three cases that were *Tp*-PCR positive but had negative serology within the window period, two cases appear to have positive serology collected after the window period (**Supplementary Table 2**). Two males had no prior syphilis serology and negative serology during the window period but subsequent testing demonstrated positive EIA and INNO-LIA with RPR dilutions of 1:512 after 50 days in one case and 1:1 after 366 days in the other. Neither patient had additional follow-up serology. A third male had negative syphilis testing prior to and within the window period but was lost to further follow-up serology.

### Syphilis PCR (*Tp*-PCR) Testing Characteristics

Estimated from all tests – collected from discrete lesions at independent timepoints – *Tp*-PCR had a sensitivity and specificity of 54.8% (49.0-60.5) and 99.9% (99.8-100.0) respectively. The positive and negative LRs were calculated to be 705 (226–2194) and 0.5 (0.4-0.5), respectively. PPV for *Tp*-PCR completed on all lesions was estimated at 98.2% (94.9-99.6) while the NPV was estimated at 96.6% (96.0-97.1). Using provincial reported rates (4.7 per 100,000 persons; Alberta Health), the PPV and NPV estimates for *Tp*-PCR diagnosis of an anogenital lesion were 0.5% (0.2-1.5) and 100.0% (100.0-100.0), respectively (Wellness AH, 2012). When HSV1 and HSV2 positive samples were excluded, there were no significant differences in test characteristics (**Table 2**).

**Table 2:** Test Characteristics for *Tp*-PCR among unique patients and independent patient cases with expert clinical suspicion of primary syphilis for all anogenital ulcers sampled and HSV1/2 negative anogenital ulcers sampled.

All Anogenital Ulcers		HSV1/2 Negative Anogenital Ulcers	
One Sample per Patient *n=3,600*		One Sample per Patient *n=2,068*	
Sensitivity	49.3 (42.6-56.1)	Sensitivity	49.3 (42.3-56.3)
Specificity	99.9 (99.7-100)	Specificity	99.8 (99.5-100)
PPV	97.4 (92.5-99.5)	PPV	97.1 (91.9-99.4)
corr. for pop. prev	0.4 (0.1-1.2)	corr. for pop. prev	0.2 (0.1-0.7)
NPV	96.7 (96.1-97.3)	NPV	94.7 (93.6-95.6)
corr. for pop. prev	100.0 (100.0-100.0)	corr. for pop. prev	100 (100–100)
LR+	555 (178–1733)	LR+	306 (98–955)
LR-	0.5 (0.4-0.6)	LR-	0.5 (0.4-0.6)
All Samples *n=4,157*		All Samples *n=2,370*	
Sensitivity	54.8 (49.0-60.5)	Sensitivity	53.7 (47.6-59.7)
Specificity	99.9 (99.8-100.0)	Specificity	99.9 (99.6-100.0)
PPV	98.2 (94.9-99.6)	PPV	98.1 (89.7-100)
PPV *(provincial)	0.5 (0.2-1.5)	PPV *(provincial)	0.3 (0.1-0.8)
NPV	96.6 (96.0-97.1)	NPV	18.2 (8.2-32.7)
PPV *(provincial)	100 (100–100)	PPV *(provincial)	100 (100–100)
LR+	705 (226–2194)	LR+	375 (121–1169)
LR-	0.5 (0.4-0.5)	LR-	0.5 (0.4-0.5)

*(provincial) – estimated value using reported rate of primary syphilis cases in Alberta over the study period (2008–2014).

Estimated from the first, discrete sample collected among the 3,600 patients, *Tp*-PCR had a sensitivity of 49.3% (95% CI; 42.6-56.1) and specificity of 99.9% (99.7-100.0) (**Table 2**). Positive and negative LRs were estimated at 555 (178-1733) and 0.4 (0.1-0.7), respectively. PPV and NPV were calculated from the test population as 97.4% (92.5-99.5) and 96.7% (96.1-97.3), respectively. When reported rates of primary syphilis within the Alberta population (4.7 per 100,000 persons) were used, the estimated PPV was 0.4% (0.1-1.2) and NPV was 100.0 (100.0-100.0) (Wellness AH, 2012). Excluding samples positive for HSV1 and HSV2, *Tp*-PCR had comparable test characteristics (**Table 2**).

## Discussion

This study confirms the high specificity (99.9%) and LR+ (>500) of the *Tp*-PCR test collected from anogenital chancres of 3,600 patients. However, the sensitivity (49.3%) and LR- (0.5) appear inadequate for its use in ruling out syphilis. In our study population, the PPV and NPV are over 95% but when considering the reported rates of primary syphilis in Alberta, the PPV is significantly reduced to less than 1%. Exclusion of HSV PCR positive samples in our secondary analysis was performed as these two diagnoses account for an overwhelming majority of anogenital lesions and did not affect the estimated test characteristics as positivity of both *Tp*-PCR and HSV1 or HSV2 occurred infrequently.

Diagnostic testing for syphilis continues to be a challenge. Serology has low performance characteristics and requires interpretation of multiple tests, whereas molecular testing can often provide a yes-no answer that is easily reported. Development of a *Tp*-PCR test has enabled improvements in the diagnosis of symptomatic primary syphilis, but evidence is limited to small studies of highly selected patients. Our study results support the continued use of a *Tp*-PCR test in clinical practice to confirm the diagnosis of primary syphilitic chancre given the known, imperfect performance of serology and in rare, but important, instances where seroconversion is delayed. While all patients, regardless of results, are likely to have been treated empirically for syphilis at the time of testing, the rapid and accurate results obtained allow public health officials to prioritize contact tracing as a method of reducing transmission.

Previous studies have varied widely in their characterization of *Tp*-PCR performance (Gayet-Ageron et al., 2013). Original development and validation studies concluded the test had high sensitivity, specificity, and negative predictive value (Leslie et al., 2007; Gayet-Ageron et al., 2009). Similar to this study’s estimates, pooling of previous small and highly selective studies demonstrates molecular testing with *Tp*-PCR has high specificity, LR+ and LR- (Gayet-Ageron et al., 2013). This pooled estimate of *Tp*-PCR test characteristics suggests modest screening capabilities with 78.4% sensitivity for primary syphilis lesions in studies they determined to have adequate reference testing (Gayet-Ageron et al., 2013). Interestingly, the pooled sensitivity for primary syphilitic ulcers among studies determined to have inadequate reference testing is estimated at 42.4% – a value much closer to the estimate of this larger and real-world study that did have sufficient reference testing (Gayet-Ageron et al., 2013). With exception of the pooled specificity from studies with adequate reference testing, the heterogeneity among all other pooled estimates is high and suggests that the numerous small studies vary considerably in their ability to determine the *Tp*-PCR characteristics (Gayet-Ageron et al., 2013). Unsurprisingly, the PPV of the *Tp*-PCR testing for primary syphilis appears to be lower than previously reported when calculated using the population prevalence (Gayet-Ageron et al., 2015). Even with a worsening epidemic, the prevalence of primary syphilis within the general population remains modest and the low positive predictive value is expected. While our study did not specifically select the population tested, the typical patient presenting to provincial STI clinics is at higher risk for syphilis (e.g. seeking anonymous testing, high proportion of male patients who have sex with men). Therefore, in the general clinical setting, the PPV of the test may actually be closer to that of the uncorrected (result-specific) estimate and of previous estimates and the test itself remains useful in the hands of experienced STI clinicians.

While our estimates of several *Tp*-PCR test characteristics (sensitivity, LR+ and LR-) are consistent with the literature, estimated sensitivity is not. Molecular testing is subject to variability of several factors, some of which are independent of the test assay itself. Perhaps the most significant of these external factors are specimen collection and handling (Yang and Rothman, 2004). While collection and handling of specimens was outside our control in this retrospective analysis, specimens in the STI clinics would have been collected by trained and experienced clinical specialists and accredited laboratory standards would have been practiced and largely negated this concern. Thus, there is increased probability that *Tp*-PCR sensitivity is lower than previously stated and its power lies in testing and confirming syphilis in patients presenting with anogenital ulcers resembling a syphilitic chancre

Limitations in our analysis include the lack of a true gold standard test as serology is limited by seroconversion and interpretation of titers. Dark field microscopy, a historic gold standard, was not used in our study as it is not routinely performed on all samples, even in the STI clinics, due to constraints with timely slide preparation, user competence, and high bacterial load required within the ulcer. Additionally, a retrospective analysis is limited by the data collection and heterogeneity of potentially unknown clinical processes. Therefore, our study was limited to STI clinics with highly trained staff and standardized protocols. Unfortunately, our study was limited to laboratory data thus clinical data from patient charts including final syphilis staging as per STI physicians, sexual behavior, and lesion location could not be thoroughly analyzed. However, lab data is sufficient to make accurate conclusions on overall testing characteristics. Lastly, while our study sampled a larger population than previous assessments of *Tp*-PCR, the low positivity rate could also have limited our assessment of test characteristics. Previous appraisals of *Tp*-PCR were done in high-risk populations with positive test rates greater than 10% due to inclusion of high-risk groups such as persons living with HIV or men who have sex with men (Gayet-Ageron et al., 2009; Gayet-Ageron et al., 2013). Our study population had a positive test rate of approximately 3,000 per 100,000 due to our use of STI clinic patients who were at much higher prevalence than Alberta’s population which had a reported rate of 3.3 per 100,000 over the study period (Wellness AH, 2019). Use of the STI clinic population was necessary to ensure clinical accuracy and the infrequent use of *Tp*-PCR test outside this setting. Despite sampling from these specialty clinics, our study still provides a population-based estimate of diverse and otherwise unselected population which reflects a real-world setting for which the test would be used when compared to previous studies that assessed specific sub-populations with the highest risk of syphilis.

In conclusion, molecular testing using *Tp*-PCR for primary syphilis appears to be a highly specific test with low sensitivity that is, therefore, most useful in confirming the diagnosis rather than as a screening test. Concurrent testing with syphilis serology remains necessary to ensure all cases are identified to manage the worsening epidemic and further work is required for the development of superior diagnostic assays that are both sensitive and specific.

## Data Availability Statement

The data analyzed in this study is subject to the following licenses/restrictions: Personal health information accessed through CHREB approval and stored according to internal data security policies. Requests to access these datasets should be directed to Caley Shukalek, cbshukal@ucalgary.ca.

## Ethics Statement

The studies involving human participants were reviewed and approved by Conjoint Health Research Ethics Board (CHREB) Alberta Health Services and University of Calgary (REB14-2126). Written informed consent for participation was not required for this study in accordance with the national legislation and the institutional requirements.

## Author Contributions

CS was involved in all components of the study including design, ethics submission, data cleaning, analysis, and interpretation, and manuscript preparation. KF and RS supervised the study through all stages including design, ethics, analysis, interpretation and manuscript preparation. BL and SF were involved in study design, data acquisition, and manuscript preparation. All authors contributed to the article and approved the submitted version.

## Conflict of Interest

The authors declare that the research was conducted in the absence of any commercial or financial relationships that could be construed as a potential conflict of interest.
