# Supplementary material for: Comparative Analysis of Molecular and Serologic Testing for Primary Syphilis: A Population-Based Cohort Study
Source: Front Cell Infect Microbiol. 2021 Apr 23;11:579660. doi: 10.3389/fcimb.2021.579660 (PMC8103196; doi:10.3389/fcimb.2021.579660)
Supplement: Supplementary file 1 [file DataSheet_1.pdf]

Comparative analysis of molecular and serologic testing for primary syphilis: a population-based cohort study

SUPPLEMENTARY DATA

Supplementary Table 1A: *Tp*-PCR results of anogenital lesions compared to syphilis serology for each unique adult patient.

		PCRpos		
		Positive	Negative	Total
Serology	Positive	111	114	225
	Negative	3	3,372	3,375
	Total	114	3,486	3,600

Supplementary Table 1B: Tp-PCR results of anogenital lesions compared to syphilis serology for each unique adult patient, excluding samples positive for HSV1 or HSV2

		PCRpos		
		Positive	Negative	Total
Serology	Positive	102	105	207
	Negative	3	1,858	1,861
	Total	105	1,963	2,068

Supplementary Table 1C: Tp-PCR results of anogenital lesions compared to syphilis serology for each adult patient encounter.

		PCRpos		
		Positive	Negative	Total
Serology	Positive	165	136	301
	Negative	3	3,853	3,856
	Total	168	3,989	4,157

Supplementary Table 1D: *Tp*-PCR results of anogenital lesions compared to syphilis serology for each unique adult patient.

		PCRpos		
		Positive	Negative	Total
Serology	Positive	146	126	272
	Negative	3	2,095	2,098
	Total	149	2,221	2,370

Comparative analysis of molecular and serologic testing for primary syphilis: a population-based cohort study

Supplementary Table 2: Summary of *Tp*-PCR Positive But Syphilis Serology Negative Cases

		More Than 7-Days **Prior** to Window Period			Within Window Period			More Than 28-Days **After** the Window Period			Comments
	Tp-PCR	EIA	RPR	INNO-LIA	EIA	RPR	INNO-LIA	EIA	RPR	INNO-LIA	
Male 1	+	**?**	**?**	**?**	-	N/A	N/A	+	1:512	+	Repeat collection outside of window period (+50 days); lost to follow-up
Male 2	+	-	**N/A**	**N/A**	-	N/A	N/A	**?**	**?**	**?**	No follow-up testing available
Male 3	+	**?**	**?**	**?**	-	N/A	N/A	+	1:1	**?**	Repeat collection outside of window period (+366 days); missing INNO-LIA result
